# Charcot stage 0: A review and consideratons for making the correct diagnosis early

**DOI:** 10.1186/s40842-015-0018-0

**Published:** 2015-12-18

**Authors:** Crystal Holmes, Brian Schmidt, Michael Munson, James S. Wrobel

**Affiliations:** The Department of Internal Medicine, The University of Michigan Medical School, Metabolism, Endocrinology & Diabetes, Domino’s Farms, Lobby C, Suite 1300, 24 Frank Lloyd Wright Drive, PO Box 451, Ann Arbor, MI 48106-0451 USA

**Keywords:** Charcot neuroarthropathy diagnosis, Stage 0 Charcot, Differential diagnosis of Charcot foot, Early diagnosis of Charcot neuroarthropathy, Osteoarthropathy, Prodromal, Natural history, Charcot foot

## Abstract

Charcot neuropathic osteoarthropathy (CN) is a rare disease (NIDDK, NIH Summary Report Charcot Workshop, 2008) that causes significant morbidity and mortality for affected patients. The disease can result in severe deformities of the foot and ankle that contribute to the development of ulcerations and amputations. Medical advances have failed to find ways to stop the progression of the disease. However, it is known that early detection of the CN has a substantial impact on patient outcomes. CN in the earliest stage is very difficult to recognize and differentiate from other similar presenting diseases. We intend to outline clinical considerations practitioners can use when evaluating a patient with early stage suspected CN.

## Background

The development of Charcot neuropathic osteoarthropathy (CN) which is rare [1] in the foot and/or ankle can lead to both structural and functional abnormalities resulting in ulcerations. Once ulcerations occur there is a higher risk for amputation. CN is also an independent risk factor for mortality [2, 3] (Table 1). It has been demonstrated CN diagnosis can be missed by referring physicians 95 % of the time prior to referral to a foot specialist [4]. CN that is identified after 8 weeks can have complications such as deformity at a rate of 67 %. CN that is identified within 4 weeks of onset has a complication rate of only 14 % [4]. Therefore, clinicians who make the diagnosis of CN early can have a great impact on the morbidity and mortality outcome of their patients with the disease.

**Table 1:** Characteristic data and analysis for patients with undetected early Charcot neuroarthropathy

	Group I	Group II	Mann–Whitney *U* test	*P*-value
n	7	15		
Age	62.3	53.5		
Follow up (Weeks)	49.9 +/− 21.7	114.4 +/− 58.8		
Time to correct diagnosis (weeks)	4.1 +/− 0.7	8.7 +/− 6.8	24.5	0.0262
Time from stage 0 to active Charcot (weeks)		10.9 +/− 7.5		
Complications:	14.30 %	66.70 %	25	0.0287
Ulceration	1	6		
Cellulitis		3		
Wound Dehiscence		2		
Septic Non-Union/Osteomyelitis		1		
Hardware Complication		1		
Tibial Fracture		1		
Solid Organ Transplantation		4		
Joint Location Involvement:	-	-	-	-
Forefoot		0		
Midfoot		12		
Hindfoot		5		
Ankle		5		
Multiple		5		

*Group I includes patients who did not progress to active Charcot foot

*Group II includes patients who did progress to active Charcot foot

Adapted from Wukich et al [4]. Characteristics from a population of people with early Charcot foot that either progressed to active CN or did not. The Group (Group II) who progressed to active CN endured a significant difference in overall complications and were diagnosed with CN much later than the group who did not progress to active Charcot foot (Group I)

This article will attempt to outline processes that clinicians can use to diagnose CN when it is in its early stage and future consideration for diagnoses targets.

## Main text

Charcot neuropathic osteoarthropathy can be missed by 95 % of providers prior to foot specialist referral [4]. Because Endocrinology uniquely situates the physician with many patient encounters involving patients with diabetes mellitus, it is critical for the specialty to be well aware of the signs and symptoms of Stage 0 CN. Stage 0 CN, a prodromal state of the disease, occurs when a foot demonstrates changes including redness, swelling, warmth, and pain, signs typically representing inflammation, in the neuropathic patient. These signs and symptoms are antecedent to foot architecture breakdown, seen in the later stages of CN. One of the most widely used CN classification system was proposed by Eichenholtz [5]. Stage 1 represents development, characterized by osseous debris, fragmentation, disruption, and dislocation seen of involved joints. In Stage 2, also known as the stage of coalescence, sclerosis, absorption of fine debris, and fusion of most large osseous fragments is seen. Lastly, in stage 3, the reconstruction and reconstitution stage, sclerosis becomes less, the major fragments are rounded and there is attempt at reformation of joint architecture [5]. Unfortunately, this classification system did not attempt to describe the prodromal phase and misses the earliest inflammatory phase.

Conducting a search in PubMed/NCBI, Google Scholar, and Cochrane Databases for Stage 0 CN symptoms reveals a paucity of published studies on the specific subject. Shibata [6], and later Sella [7], were the first to describe changes associated with Stage 0 CN in leprotic and diabetic patients, respectively. As far as we are aware, no paper describes the methodology to accurately diagnose Stage 0 CN patients and refer to a foot specialist. This lack of discussion about Stage 0 CN gives reason to present up to date information about CN to those most likely to encounter the Stage 0 CN patient. We also stress that CN needs to be included in the differential diagnosis for neuropathic patients that present with newly onset red, hot, swollen foot because if it not, it often goes misdiagnosed [8–11]. In this review article, we will further define stage 0 and give the practicing endocrinologist pragmatic tools to appropriately identify CN and refer to a foot specialist for further management.

## Epidemiology and pathophysiology

Charcot neuropathic osteoarthropathy is a rare destructive disease with a prevalence of 0.1 %-0.9 % [12–14] (Table 2). Although the true etiology of CN is unknown, it is accepted that neuropathy precedes the disease. In a conceptual model proposed by Koeck, et al [15]**,** important components include neurotrophic, microtrauma, and neurovascular effects [15] including a stage of pro-inflammatory cytokine activity of with pro-inflammatory cytokines, such as elevated Tissue Necrosis Factor alpha [16]. (TNF α) and Receptor Activator Nuclear Factor K ligand (RANKL) [8] (Table 3).

**Table 2:** Incidence of Charcot Neuroarthropathy in Patients with Diabetes

Reference	No. of Cases (No. of Feet)	Reported Incidence
Sinha et al. 1971 [55]	101 (N/A)	0.1 %
Cofield et al. 1983 [56]	96 (116)	7.5–29 %
Sella et al. 1999 [7]	40 (51)	5 %
Fabrin and Holstein 2000 [13]	115 (140)	0.3 %/year
Sanders et al. 2001 [57]	N/A	0.1–7.5 %
Rajbhandari 2002 [58]	N/A	0.1–0.4 %
Hartemann-Heurtier et al. 2002 [59]	N/A	0.2–3 %
Lavery et al. 2003 [14]	N/A	0.0085 %/year

Adapted from Frykberg, R and Belczyk, R [17]. A brief review of the literature demonstrating the relative low incidence of CN in the overall population. There is a range of incidences reported from 0.10 to 29.00 % and seems consistent over time

**Table 3:** Etiology of CN Model Proposed by Koeck et al

	Skin (OA)	Skin (CN)	Synovium (OA)	Synovium (CN)	Bone (OA)	Bone (CN)
Substance P positive Nerve Fibers	~3.5 nerve fiber per mm^2^	~3 nerve fiber per mm^2^	~3 nerve fiber per mm^2^	~2 nerve fiber per mm^2^	~4.5 nerve fiber per mm^2^	~4 nerve fiber per mm^2^
Sympathetic Nerve Fibers	~7 nerve fiber per mm^2^	~2 nerve fiber per mm^2^	~3 nerve fiber per mm^2^	~0.5 nerve fiber per mm^2^	~1.5 nerve fiber per mm^2^	~0.25 nerve fiber per mm^2^

Density of Substance P Nerve Fibers and Sympathetic Nerve Fibers in Skin, Synovium, and Bone of Patient’s with Charcot Neuroarthropathy and Osteoarthritis

Table is adapted from Koeck et al [56]. In their study they demonstrated that the Charcot joint (synovium) demonstrates a lack of sympathetic control compared to the control sample of patients with osteoarthritic joints. Here we report the approximate mean from their study to demonstrate the difference. The p-value between synovium concentration of sympathetic nerve fibers is <0.006 and indicates a significant difference between the two conditions. It was the only difference between the two groups that was significant

## Risk factors for Charcot

**Fig. 1:**
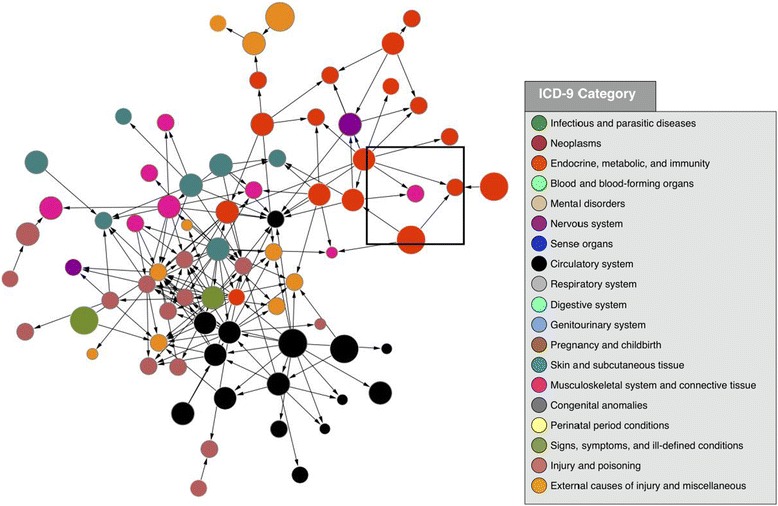
Reproduced with permission from Munson et al [21]. Association diagram showing the clinical milieu in which CN (pink node in center of square) often exists. Each node represents an ICD-9 code, with the size of the node proportional to its frequency in the overall dataset, and node colors representing high-level clinical categories (see legend). Edges between nodes represent highly significant associations. Arrowheads show temporality with preceding nodes pointing to subsequent nodes. This figure was made using the following criteria: Association *p*-value < 1.0 × 10^-176^; association odds ratio > 200; temporal p-value < 1.0 x 10^-6^. The two red nodes directly pointing to Charcot foot are related to type 2 diabetes (ICD-9 codes 250.60 and 250.90)

Clinicians can begin to develop a picture for the patient who presents with CN by being familiar with the risk factors. Risk factors for CN include, advanced age, male gender, white race, lower educational level, body mass index (BMI), duration of diabetes, peripheral neuropathy, decreased bone mineral density (BMD), and a history of pancreas and/or kidney transplant surgery [17]. Other risk factors identified using VA administrative data include: elevated HbA1c, renal failure, rheumatoid arthritis, iron deficiency anemia and obesity [2]. Petrova et al. in 2005 [18] also noted the relationship between patients with osteopenia and CN. There has also been a documented correlation between CN and patients that with end-stage kidney disease and renal transplantation [19, 20]. As a rare disease, it can be very useful to look use big data science methodologies to elucidate previously unknown risk factors for the CN. Large databases with hundreds of thousands of diabetics can be mined to identify large enough numbers with CN to better understand the epidemiology, risk factors, and management of these patients. For example, Munson and colleagues used a data mining approach to identify 710 associations of different medical conditions with CN with 111 having temporal associations with the development of CN [21] (Fig. 1).

## Amputation risk

Charcot neuropathic osteoarthropathy increases the affected patient’s risk of foot ulcer by more than 30-fold, with 63 % of persons with Charcot foot eventually developing foot ulcer [2]. Using Medicare data Wrobel and Mayfield demonstrated that diabetes increases the risk of major amputation by 10-fold [22]. According to Sohn et al. 2010, the risk of amputation in those patients with Charcot foot is 6.6 % in the community, and more than double in VA patients at 14.7 %. However, when Charcot Foot occurs with foot ulcer, the patient is at a 12-fold higher risk of amputation than patients with Charcot alone [23]. Charcot Foot has also been described to be an independent risk factor for mortality after controlling for foot ulcer and other comorbid conditions [2].

Rogers and Bevilacqua describe an amputation classification risk scheme illustrating how as Charcot deformity, ulceration and osteomyelitis approach the proximal foot and ankle, amputation risk increases [24] (Table 4).

**Table 4:**
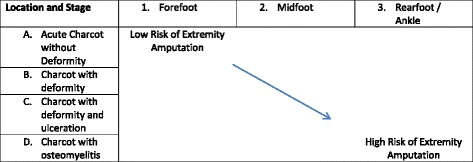
Amputation Risk Rogers & Bevilaqua

A combined anatomic and complexity classification of Charcot neuroarthropathy

Adapted from Rogers and Belivqua [60]. Using both an anatomic model combined with level of complexity, it is clearly demonstrated that as one progresses both proximal (to the right on the graph) in anatomic location and/or toward osseous involvement (down on the graph), the risk of major amputation increases

## Differential diagnosis

Frequent misdiagnosis has been reported with conditions such as cellulitis, gout, deep-vein thrombosis (DVT), osteomyelitis [9], or even osteoarthritis [8]. Clinicians must have a high index of suspension for neuropathic patients presenting with Charcot stage 0. The addition of Charcot foot to their list of differential diagnosis for patients with the classic red, hot, swollen foot may help decrease the number of missed cases. It is important to note that patients frequently present with varying degrees of swelling, warmth and redness. Minor trauma should not be dismissed. Charcot Foot may also be preceded by events of foot surgery in 22 % of cases [10] and injuries such as ankle sprains [11].

## Clinical assessment

**Table 5:** Recognition of stage 0: Sella & Barrette Staging of Charcot

Stage			Diagnosis		
0			Localized heat and midfoot swelling		
1			Localized osteoporosis, subchondral cysts, erosions, and diastasis		
2			Joint subluxations		
3			Joint dislocations		
4			Sclerosis and ultimate fusion of involved joint		
Stage	No. of Feet	Radiographs	Scans – Tc99	Scan- In/Ga	Clinical Findings
0	10	Negative	+	-	Increased heat
1	6	Cysts, erosions, diastasis	+	-	Increased heat and swelling
2	16	Joint subluxation	+	-/less +	Mild pronation
3	12	Joint dislocation	+	-/less +	Bony prominences, pronation, rocker bottom
4	7	Joint Fusions and Sclerosis	-	-	Rocker bottom, bony prominences, pronation

Staging for Charcot Foot from Sella and Barrette

Adapted from Sella and Barette [7]. A simple classification of patient with different stages of CN with associated symptomatology and clinical, radiographic, and nuclear scan findings. This study involved a group of 51 feet with diagnosed CN

A. **Recognition of Stage 0**

Another important step in identifying CN is the clinician’s ability to recognize stage zero. Most clinicians have been trained to use radiographs to screen and diagnosis the Charcot foot but waiting for radiographic changes may result in increased comorbidity for patients. Historically, clinicians refer to the Eichenholtz classification which describes three stages of Charcot using radiographs [5]. Stage I, the developmental stage; bone fragmentation, osseous debris, osseous fragmentation, and disruption or dislocation of joints were noted radiographically. Stage II, the stage of coalescence; there was sclerosis, absorption of bone fragments and fusion of most large fragments was noted to adjacent bone. Stage III, the stage of reconstruction and reconstitution; there is lessened sclerosis, remodeling and rounding of bone ends, with an attempt at reformation of joint architecture [5]. Shibata et al. described CN Stage 0 in 1990 in which was the clinical presence of swelling, erythema and warmth in the presence of normal radiographs in patients with leprotic neuroarthropathy [6]. In 1999 Sella and Barette described stage 0 in patients with CN [7] (Table 5). This prodromal phase is antecedent to foot architecture breakdown with inflammation seen clinically.

B. **Anatomic Location**

**Fig. 2:**
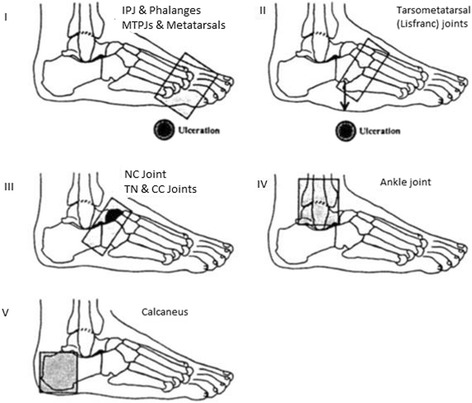
Adapted from Rogers and Frykberg [27] Staging of the Charcot foot based on anatomic location within the foot. Five anatomic patterns are represented with pattern I affecting the phalanges, IPJs, MTPJs and distal metatarsal bones with atrophic and destructive changes; pattern II affecting the tarsometatarsal joints (Lisfranc’s joint) often with ulceration at apex of collapsed cuneiforms of cuboid; pattern III affecting the naviculocuneiform, talonavicular, and calcaneocuboid joints (Chopart’s joint) with fragmentation of the NC joint and/or subluxation of the CC and TN joints, pattern IV representing the talocrural joint (Ankle joint) and subtalar joints, and pattern V representing involvement of only the calcaneal bone, and particularly avulsion of the posterior tuber of the calcaneus

Charcot neuropathic osteoarthropathy typically affects the midfoot. Several authors have described the anatomic location is associated with CN. One of the most recognized classification is the Sanders classification which it clearly highlights the midfoot as the most targeted area for Charcot development [25–27] (Fig. 2). It has been hypothesized that limited ankle joint range of motion coupled with neuropathy and obesity may predispose the mid-foot for breakdown.

C. **Temperature Gradient and Other Clinical Signs**

The clinical presentation of CN stage 0 is characterized by an edematous, erythematous warm foot. Some discussion is necessary to quantify the temperature increase. Armstrong and Lavery reported the baseline infrared dermal thermometry results for 39 patients presenting with unilateral acute Charcot foot [28] After 15 min’ rest, they found an average 8.8 ± 2.3 °F higher temperature compared to the contralateral joint of interest (JOI). In a separate study, the same team reported specific mean joint differences of 7.3 °F, 8.0 °F, and 8.8 °F for the ankle Chopart, and Lisfranc’s joint respectively [28, 29]. The temperature differences were found to correlate highly with radiographic changes [28] and with markers of bone turnover [30]. In the diabetic foot, statically measured joint risk factors may not be associated with dynamic activity [31]. Najafi and colleagues studied 15 patients with acute CN and 17 patients with diabetes-related peripheral neuropathy. At baseline the CN patients demonstrated a significant 1.84 +/− 1.3C temperature difference between the affected and unaffected foot. This difference is below the threshold for both diagnosing and treating CN [30]. While significant, this difference was less than 4.1C – 4.9C difference found by Armstrong and Lavery [32]. Following walking of 50 steps and 150 steps, the baseline temperature differences between feet increased significantly by 60 % [33]. As most bouts of activity for patients with diabetes-related peripheral neuropathy are 50 steps or less [34], dynamic temperature testing may be clinically important. CN findings are typically unilateral. Pedal pulses may be palpated in circumstances where there is not marked edema.

D. **Laboratory testing**

While there is no definitive or specific laboratory marker to diagnose CN, patients with CN may have leukocytosis, elevated hsCRP and ESR as seen with other inflammatory conditions. Hemoglobin A1C elevation of > 7 % is common [35]. In instances where CN is one of the differential diagnoses elevated uric acid levels may be necessary to determine if a patient has gout. Clinical evaluation for inflammation is paramount in diagnosis.

E. **Histology to Confirm Charcot Neuropathic Osteoarthropathy**

**Fig. 3:**
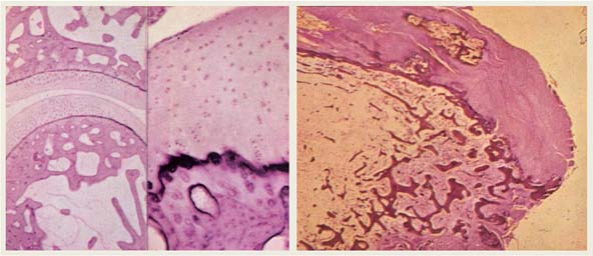
Histology Slides demonstrating histologic changes seen in patient with Charcot Neuroarthropathy [36]. The figure on the left demonstrates a normal joint; here it is a distal interphalangeal joint. Note the smooth cartilage surface, organization of the chondrocytes in regular rows, and the subchondral cancellous bone is intact. The figure on the right demonstrates a joint afflicted by CN. Note the absence of cartilage and replacement with fibro-osseous tissue. The major histologic changes are evident at the joint, as demonstrated in the normal and pathologic samples above

It is also important to remember that in circumstances where a biopsy can be performed that may be beneficial to differentiate between other disease processes such as osteomyelitis. In patients with a normal joint, the articular cartilage is smooth, chondrocytes line up in regular rolls and subchondral cancellous bone is intact. In joints affected by CN there are degenerating fibrillary remains of cartilage, absence of cartilage, and fibro osseous tissue [36]. La Fontaine et al. characterized the CN bone further [37]. These authors illustrate that CN bone histology has characteristics of reactive bone with presence of woven bone that was immature and structurally disorganized. Further the bone marrow spaces were infiltrated with hypervascular, myxoid tissue with spindle fibroblasts with an increase in the number of Howship’s lacunae and a decreased number of osteocytes. This was less than that observed in both the normal and DM groups of the study [37] (Fig. 3).

F. **Imaging Modalities used to diagnose Charcot**

Utilization of radiographs alone to determine the onset of Charcot foot is not advised. We suggest that clinicians utilize clinical judgment and consider other modalities in addition to radiographs to diagnose CN in its earliest stage. Radiographs should be performed to scrutinize later stages of CN where fragmentation, fracturing, dislocations and effusions of joints exist. A MRI is superior to a radiograph to diagnose CN Stage 0. We recognize that there are instances where clinical judgment supersedes the need for a MRI. There are also circumstances where MRI may not be the most cost-effective or patient friendly based on other disease states such as a diminished kidney function. However, MRI can detect bone marrow edema and is therefore far more sensitive and specific then a radiograph in the detection of Charcot earliest stage.

In April 2014 Chantelau and Grutzner [38] proposed a new classification for CN which includes both clinical, MRI, and radiographic as well as histologic findings. This classification system seems to be the most current in utilizing all of the current tools for diagnosing Charcot in its earliest phase and throughout the entire course of the disease recognizing both the active and inactive stages of involvement (Tables 6 and 7).

**Table 6:** Chantelau and Grutznel MRI Classification of the Charcot Foot

	Low Severity (without cortical fracture)	High Severity (with cortical fracture)
Active Arthropathy	Mild inflammation/edema No skeletal deformity X-ray is otherwise normal MRI: Abnormal with edema, microfractures and bone bruise	Severe edema/inflammation Severe Skeletal deformity Microfractures on X-ray MRI: Abnormal with edema, macrofractures and bone bruise
Inactive Arthropathy	No inflammation No skeletal deformity X-ray is otherwise normal MRI: No significant edema	No inflammation Skeletal deformity X-ray with past macrofractures MRI: No significant edema

**Table 7:** Combined Clinical symptoms, Advanced Imaging and Histopathology Classification

	Clinical Signs and Symptoms	CT and MRI features	Histopathology
Active stage, grade 0	Mild inflammation but no gross deformity	Obligatory: diffuse BMO and STO (Kiuru Grade I–III), No cortical disruption. Facultative: subchondral trabecular microfractures (bone bruise); ligament damage	Lamellar bone with active surface. Remodelling of trabeculae associated with microfractures. Marrow space replaced by loose spindle cells.
Active stage, grade 1	Severe inflammation with gross deformity, increased by unprotected walking	Obligatory: fracture(s) with cortical disruption, BMO and STO (Kiuru grade IV). Facultative: osteoarthritis, cysts, cartilage damage, osteochondrosis, joint effusion, fluid collection, bone erosion/necrosis, bone lysis, debris, bone destruction, joint luxation/subluxation, ligament damage, tenosynovitis, bone dislocation.	Increased vascularity of the marrow space, active remodelling of woven bone. Compatible with response to (impaction) fracture. Osteonecrosis. Thickened synovium, fragmented cartilage and subchondral bone, invasion of inflammatory cells and vascular elements
Inactive stage, grade 0	No inflammation, no gross deformity.	No abnormal imaging, or minimal residual BMO; subchondral sclerosis, bone cysts, osteoarthrosis, ligament damage	Sclerosis of bone characterized by broad lamellar trabeculae with collagenous replacement and a low vascularity of the marrow space
Inactive stage, grade 1	No inflammation; persistent gross deformity and possible ankylosis	Residual BMO, cortical callus (Kiuru grade IV); joint effusion, subchondral cysts, joint destruction, joint dislocation, fibrosis, osteophyte formation, bone remodelling, cartilage damage, ligament damage, bone sclerosis, ankylosis, pseudoarthrosis	Woven bone, immature and structurally disorganized, fibrosis

Adapted from Chanetelau and Gruetzner [38] classification of the Charcot foot using MRI to differentiate between high and low severity in active versus inactive CN. The second table combines clinical, MRI, and histopathologic findings in accordance with Charcot foot severity

Bone scanning can be a useful tool in differentiation of Charcot neuropathic osteoarthropathy with and without osteomyelitis. It however must be used with caution because leukocyte labeled scintigraphy does not always demonstrate changes where bony turnover is occurring. Poor sensitivity is often attributable to chronicity of infection, while poor specificity is attributable to nonspecific inflammatory changes [39]. It must be stated that in the earliest stages of Charcot, many polymorphonuclear cells (PMNs) are present with acute inflammation [40]. The scans are therefore useful early to the astute physician and can demonstrate changes in tissue activity in a day as compared to 10–14 days with standard radiographs. With the development and addition of several tags to the leukocyte and regardless of the exact understanding of the mechanism why labeled leukocytes accumulate in the uninfected Charcot neuropathic joint [41, 42] bone scanning can continue to be a useful non-invasive imaging modality to differentiate between the diagnosis of Charcot neuropathic osteoarthropathy and osteomyelitis.

Historically, ^99^technetium-MDP, gallium-67 citrate, and indium-111 played a role in the differentiation between soft tissue infection and osseous infection [43]. Gallium was used because it has high affinity toward inflammatory processes but lacked affinity for osteoblastic activity. Indium had a longer half-life than gallium and had greater specificity for infection because it depended on leukocyte chemotaxis. However, these early scintigraphy agents were not without fault because they had low specificity and were effectively useless to differentiate certain conditions [44]. Hexamethylptopyleneamine oxime *(*HMPAO or Ceretec) is commonly used today to differentiate osteomyelitis from Charcot joint. Traditionally, the HMPAO labeled leukocytes are injected into the subject and a three phase bone scan is performed at intervals as described as blood flow, blood pool, and bony turnover stages as discussed by Thakur et al [45]. Once the scan has been completed, the images are inspected at each stage for activity in the affected area. If there is positive activity at each stage, then the cellular activity is consistent with osteomyelitis. In 2001, Boc et al. demonstrated that while HMPAO was the most reliable non-invasive imaging study that could be done for differentiating osteomyelitis from Charcot foot changes, it was second to bone biopsy because false positives do occur [46]. More recently, Morbach et al. demonstrated MRI rather than plantar bone scintigraphy was superior for detection of chronic osteomyelitis with sensitivity of MRI at 100 % and for bone scintigraphy at 78.4 % [47].

The basis for the addition of sulfur colloid to a technetium bone scan lies in that both sulfur colloid and leukocytes have an affinity toward cellular activity in the bone marrow. Accordingly, this should be done in concert with another labeled leukocyte scan, but it is described as being done one hour after completion of the three phase bone scan [44]. Leukocytes are attracted to areas of infection and sulfur colloid is not [48]. More important is that similar patterns are demonstrated on image comparison between healthy individuals and those with abnormalities when using the sulfur colloid scan. When a patient has osteomyelitis, there is a localized increase in leukocyte uptake observed along with suppression of sulfur colloid [49]. By utilizing this inherent advantage, when there is an edematous foot with a question of infection or a Charcot event, the addition of sulfur colloid to a technetium bone scan can aide in the differentiation.

## What does the evidence tell us as it relates to diagnosing Charcot neuropathic osteoarthropathy?

In 2003 Milne et al. reviewed [50] the level of evidence as it relates to CN treatment and diagnoses. Magnetic resonance imaging has a level III; nuclear medicine has a level of evidence IV (Table 8). FDG-PET has a level of evidence IV and bone biopsy has a level of evidence expert opinion. We believe this indicates the need to conduct randomized clinical trials in patients with Charcot foot.

**Table 8:** Levels of Evidence (Miline) [50]

Level of Evidence	Definition
I	A systematic review of level II studies
II	A randomized controlled trial
III	A pseudorandomized controlled trial (alternate allocation, etc.)
III-2	A comparative study with concurrent controls (cohort, case–control)
III-3	A comparative study without concurrent control (historical cohort)
IV	Case Series
EO	Expert Opinion – where evidence was absent or unreliable and advice was formulated based on clinical judgement of experts in the fields

The ADA consensus report performed by Rogers and associates [3] illustrate an algorithm for diagnosis of CN. Their algorithm clearly outlines the clinician starting with a clinical suspicion of CN and navigating through x-rays, MRI or other nuclear imaging based on the patient’s clinical findings, diagnostic results followed by the response to treatment.

## Future considerations

The pathogenesis of Charcot neuropathic osteoarthropathy (CN) is multifactorial and not entirely determined. Neuropathy, inflammation, obesity, and hyperglycemia all play major roles in CN development and progression. Peripheral neuropathy includes sensory, motor, autonomic, and neurogenic peptide dysregulation [51]. As a result, the patient does not necessarily perceive traumatic events that could lead to areas of increase pressure and potential sites of breakdown [52, 53]. This perpetuates the inflammatory cycle, leading to a vicious cycle that intimately affects bony turnover [54].

**Fig. 4:**
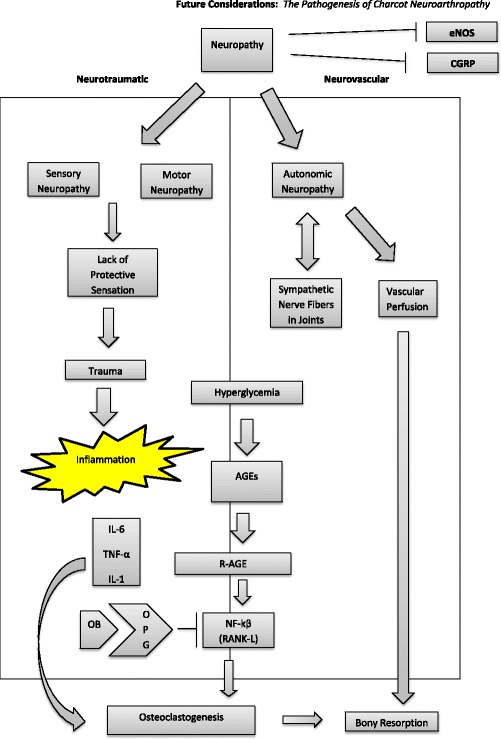
The pathogenesis of CN is multifactorial and not entirely determined. Neuropathy, inflammation, and hyperglycemia all play major roles in CN development and progression. Peripheral neuropathy includes sensory, motor, autonomic, and neurogenic peptide dysregulation [52]. As a result; the patient does not necessarily perceive traumatic events that could lead to areas of increase pressure and potential sites of breakdown [53]. This perpetuates the inflammatory cycle, leading to a vicious cycle that intimately affects bony turnover [54]. The bony turnover is also regulated by hyperglycemia which is shown to increase advanced glycation end products (AGEs). AGEs lead to an increase in receptor for AGEs (RAGE). The increase in RAGE leads to an increase in RANK-L which promotes osteoclastogenesis [55]. Finally, newer evidence demonstrates the Charcot neuroarthropathic joint itself is lacking sympathetic control and may allow for increase perfusion to the area [32]. This may also disturb the bony turnover ratio leading to weakened demineralized bone

The bony turnover is also regulated by hyperglycemia which is shown to increase advanced glycation end products (AGEs). AGEs lead to an increase in receptor for AGEs (RAGE). The increase in RAGE leads to an increase in RANK-L which promotes osteoclastogenesis [15]. Finally, newer evidence demonstrates the CN joint itself is lacking sympathetic control and may allow for increase perfusion to the area [18]. This may also disturb the bony turnover ratio leading to weakened demineralized bone (Fig. 4).

## Conclusion

**Fig. 5:**
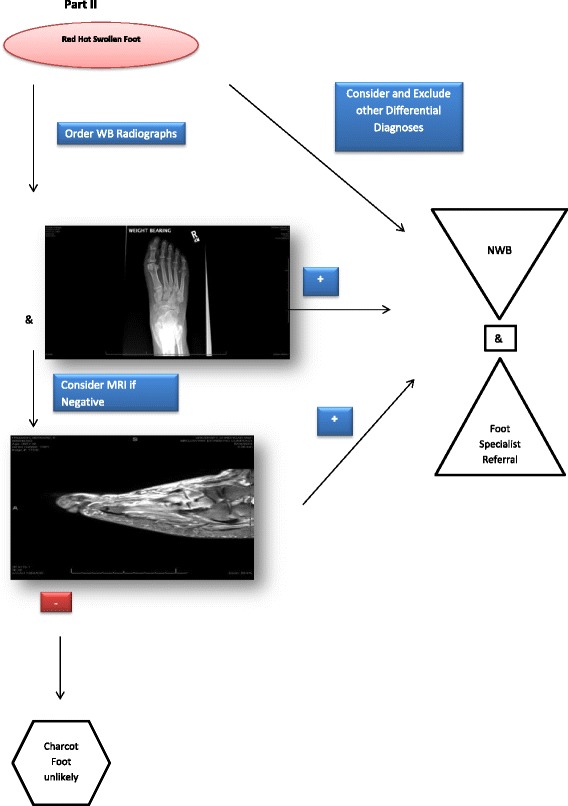
CN Stage 0 Evaluation Algorithm Part II

In summary it is important to note that each clinician who evaluates a patient with CN has the opportunity to substantially change the outcome in their patients. They should start by having a high index of suspicion which cannot occur unless the clinician adds CN to their list of differential diagnosis when evaluating a patient with neuropathy, diabetes, and other listed risk factors. The ability for the astute clinician to recognize stage zero is also pivotal as in terms of deformity prevention and long term outcomes (Table 9 & Fig. 5.). Offload patient during evaluation process to prevent structural damage. A wheelchair, crutches, or a walking boot are all suitable options. Referral to foot specialists and implementation of diagnostic tools in a timely fashion such as dermal temperatures in joint regions of interest, radiographs, MRI, bone scan and other modalities based on their facilities capabilities as early as possible is also crucial. We hope that in the future the diagnosis of Charcot neuropathic osteoarthropathy will be simple, but until that time surveillance and ongoing discussions about how we can improve diagnostic strategies is the key to limb preservation.

**Table 9:** CN Stage 0 Evaluation Algorithm Part I

Differential Diagnoses	Erythema	Edema	Warmth	Pain	Skin Break	Temperature Difference (>4C)	Peripheral Neuropathy
Gout	+	+/−	+	++	-	-	-
DVT	+	+	+	+/−	-	-	-
Cellulitis	+	+	+	+	+	-	+/−
CN (stage 0)	+	+	+	+/−	-	+	+

## Abbreviations

CN: Charcot neuropathic osteoarthropathy
